# Supplementary material for: Associations between Exposure to Perfluoroalkyl Substances with Subsequent Body Composition and Glycemic Responses to Bariatric Surgery
Source: medRxiv. 2026 Apr 1:2026.03.30.26349786. Preprint. [Version 1] doi: 10.64898/2026.03.30.26349786 (PMC13060460; doi:10.64898/2026.03.30.26349786)
Supplement: 1 [file NIHPP2026.03.30.26349786V1-supplement-1.pdf]

medRxiv preprint doi: https://doi.org/10.64898/2026.03.30.26349786; this version posted April 1, 2026. The copyright holder for this preprint (which was not certified by peer review) is the author/funder, who has granted medRxiv a license to display the preprint in perpetuity.
It is made available under a CC-BY 4.0 International license .

Supplementary Material

Table S1. Longitudinal Plasma Concentrations of PFAS (ng/mL)

PFAS Species	n	Time Point	Geometric Mean [95% CI]	SD	50th %	75th %	n (%) Below LOD
PFHxS	32	Baseline	2.353 [1.660, 3.334]	2.630	1.952	4.391	0 (0%)
	22	Follow Up	0.904 [0.466, 1.753]	4.455	0.855	2.813	1 (4.54%)
	22	Average	1.449 [0.853, 2.464]	3.309	1.327	3.187	0 (0%)
PFNA	32	Baseline	1.207 [0.907, 1.606]	2.210	1.164	1.551	0 (0%)
	22	Follow Up	0.898 [0.604, 1.335]	2.445	0.969	1.192	1 (4.54%)
	22	Average	1.024 [0.750, 1.398]	2.019	0.952	1.339	0 (0%)
PFOS	32	Baseline	7.884 [5.814, 10.69]	2.328	6.980	12.87	0 (0%)
	22	Follow Up	5.619 [3.607, 8.753]	2.718	5.250	8.150	0 (0%)
	22	Average	6.169 [4.130, 9.217]	2.473	5.129	8.731	0 (0%)

Wilcoxon signed-rank tests for paired pre- and postoperative samples. LOD = limit of detection. n = 22 for all compounds.

medRxiv preprint doi: https://doi.org/10.64898/2026.03.30.26349786; this version posted April 1, 2026. The copyright holder for this preprint (which was not certified by peer review) is the author/funder, who has granted medRxiv a license to display the preprint in perpetuity.
It is made available under a CC-BY 4.0 International license .

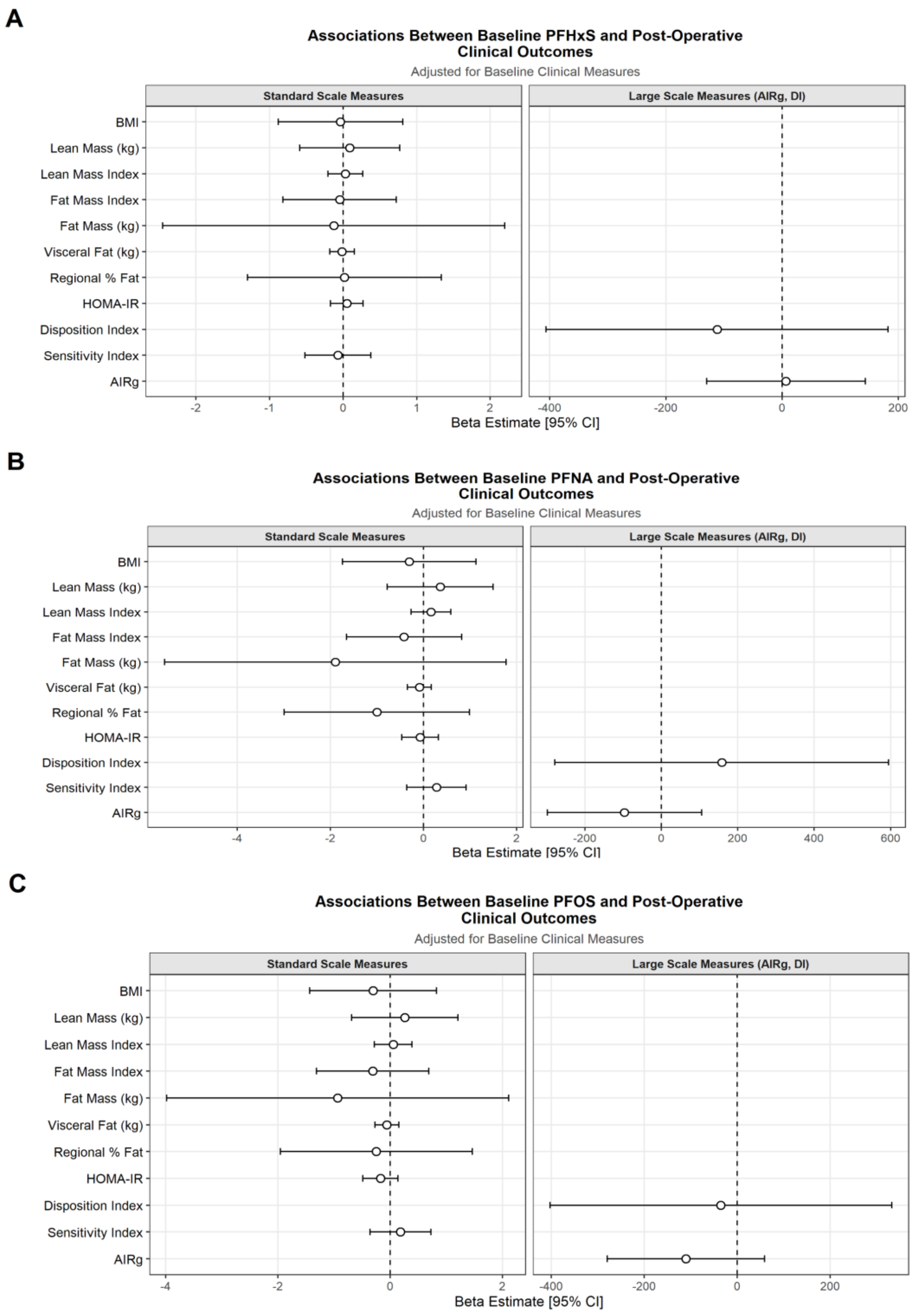

Figure S1. Baseline PFAS ANCOVA with Postoperative Clinical Outcomes. Forest plots display ANCOVA-derived beta estimates from models including baseline PFAS and the corresponding baseline clinical value for (A) PFHxS, (B) PFNA, and (C) PFOS (n=26 for all outcomes except n=23 for AIRg, DI, and SI), respectively. No statistically significant associations or consistent patterns were detected across the analyzed clinical outcomes. AIRg = Acute Insulin Response to Glucose. DI = Disposition Index. HOMA-IR = Homeostatic Model Assessment of Insulin Resistance. SI = Sensitivity Index.
